# Supplementary material for: Determination of key functional structures of an amorphous VHL-based SMARCA2 PROTAC
Source: Nat Commun. 2025 Nov 3;16:9694. doi: 10.1038/s41467-025-65478-5 (PMC12583731; doi:10.1038/s41467-025-65478-5)
Supplement: Supplementary file 1 — Supplementary Information [file 41467_2025_65478_MOESM1_ESM.pdf]

Supplementary Information

Determination of key functional structures of an amorphous VHL-based SMARCA2 PROTAC

Daria Torodii,[1,#] Jacob B. Holmes,[1,2,#] Manuel Cordova,[1,2] Pinelopi Moutzouri,[1] Lotte van Beek,[3] Fredrik Edfeldt,[4] Erik Malmerberg,[5] Stig D. Friis,[5] Johan R. Johansson,[5] Alexander G. Milbradt,[3] Sten O. Nilsson Lill,[6] Benjamin Malfait,[7] Staffan Schantz,[7] Lyndon Emsley[1,2]

[1] Institut des Sciences et Ingénierie Chimiques, École Polytechnique Fédérale de Lausanne (EPFL), CH-1015 Lausanne, Switzerland

[2] National Centre for Computational Design and Discovery of Novel Materials MARVEL, École Polytechnique Fédérale de Lausanne (EPFL), Lausanne, Switzerland

[3] Protein Science, Structure & Biophysics, Discovery Sciences, AstraZeneca R&D, Cambridge, UK

[4] Mechanistic and Structural Biology, Discovery Sciences, R&D, AstraZeneca, Gothenburg, Sweden

[5] Medicinal Chemistry, Research and Early Development Cardiovascular, Renal and Metabolism, BioPharmaceuticals R&D, AstraZeneca, Gothenburg SE-431 83, Sweden

[6] Data Science & Modelling, Pharmaceutical Sciences, R&D, AstraZeneca, Gothenburg, Sweden

[7] Oral Product Development, Pharmaceutical Technology & Development, Operations, AstraZeneca, Gothenburg, Sweden

[#] These authors contributed equally to this work

Raw data: All data are available from [https://doi.org/10.24435/materialscloud:d0-ck] under the license CC-BY-4.0 (Creative Commons Attribution-ShareAlike 4.0 International).

Table of Contents

1. Experimental Details 3
2. Additional NMR data 6
3. Assignment and fitting details 10
4. Additional histograms of the dihedral angles and relative cluster formation energies 16
5. Computational details for the MD simulations 21
6. Synthesis of PROTAC 2 22
7. Solution ^{1}H-, ^{13}C- and ^{19}F-NMR, HRMS and X-ray diffraction data for PROTAC 2 24

1. Experimental Details

All data and codes used are available from [https://doi.org/10.24435/materialscloud:d0-ck]. The exact pulse sequences and full parameter sets used are available with the raw data.
The sample was packed in 0.7 and 3.2 mm rotors after being crushed with a mortar and pestle. All the experiments at 100 kHz MAS were acquired at room temperature using a Bruker 0.7 mm room temperature HCN CP-MAS probe at a magnetic field of 21.1 T corresponding to a ^{1}H frequency of 900 MHz. The temperature was kept constant using a VT flow of 285 K. All the experiments at 8 and 10 kHz MAS were acquired using a Bruker 3.2 mm H/X/Y LTMAS probe connected through a corrugated waveguide to a 264 GHz klystron or a 263 GHz gyrotron microwave source at 9.39 T (^{13}C Larmor frequency of 101 MHz). A States-TPPI acquisition scheme was used in all 2D experiments to obtain phase-sensitive two-dimensional spectra. All spectra were phase and baseline corrected. An exponential window function of 200 and 300 Hz in the direct and indirect dimension, respectively, was applied prior to Fourier transformation of the hCH spectra.

Table S1. Experimental details for PROTAC 2

Experiment	MAS rate (kHz)	^{1}H 90° RF amplitude (kHz)	CP contact time, ms	recycle delay(s)	Number of FID points	SW (kHz)	Size of real spectrum		Number of scans
1D ^{1}H echo	100	285	286	-	2.1	8192	227	16384	4
hCH long-range	100	285	250	5 (direct CP) & 5 (back CP)	2.1	1024 (F2) / 48 (F1)	200 (F2) / 50 (F1)	8192 (F2) / 256 (F1)	2048
hCH short-range	100	285	286	0.5 (direct CP) & 0.25 (back CP)	2.1	512 (F2) / 128 (F1)	91 (F2) / 45 (F1)	4096 (F2) / 1024 (F1)	1024
^{1}H-^{1}H BABA	100	285	286	-	2.1	2048 (F2) / 400 (F1)	227 (F2) / 50 (F1)	8192 (F2) / 1024 (F1)	16
^{1}H-^{13}C CP powder dissolved in 16mM TEKPol TCE	10	100	100	2	3	1024	50	2048	32
^{19}F-^{13}C CP as received powder	11	300	95	3	5	804	30	2048	8192
Cq-edited CP powder dissolved in 16mM TEKPol TCE	10	100	100	2	3	1024	50	2048	64
^{13}C{^{14}N} RESPDOR (attached ^{14}N test) powder dissolved in 16mM TEKPol TCE	10	100	96	2	3.9	1024	50	4096	8192
^{15}N CP powder dissolved in 20mM TEKPol TCE-d_2	10	100	93	5	4.4	1626	41	4096	4096
^{1}H-^{15}N DUMBO-HETCOR powder impregnated with 12mM AMUPol in DNPj	10	100	91	1.5	5	4096 (F2) / 128 (F1)	100 (F2) / 23 (F1)	8192 (F2) / 512 (F1)	128

Table S2. Experimental details for the VT measurements of PROTAC 2 at 100 kHz MAS.

Experiment	T_{set} (K)	T_{sample} (K)	^{1}H 90° RF amplitude (kHz)	recycle delay(s)	Number of FID points	SW (kHz)	Size of real spectrum	Number of scans
1D ^{1}H echo	263	259	286	1	4096	227	8192	4
1D ^{1}H echo	265	262	286	1	4096	227	8192	4
1D ^{1}H echo	270	270	286	1	4096	227	8192	4
1D ^{1}H echo	275	277.5	286	1	4096	227	8192	4
1D ^{1}H echo	280	285	286	1	4096	227	8192	4
1D ^{1}H echo	285	293	286	1	4096	227	8192	4
1D ^{1}H echo	290	301	286	1	4096	227	8192	4
1D ^{1}H echo	295	308.5	286	1	4096	227	8192	4
1D ^{1}H echo	300	313	286	1	4096	227	8192	4
1D ^{1}H echo	305	321	286	1	4096	227	8192	4
1D ^{1}H echo	310	329	286	1	4096	227	8192	4

All the solution-state spectra of PROTAC 2 dissolved in DMSO-d_6 except the 1D ^{1}H VT set were acquired at room temperature using a Bruker 5 mm cryoprobe at a magnetic field of 18.8 T corresponding to a ^{1}H frequency of 800 MHz. The temperature was kept constant using a VT flow of 298 K. The 2D HSQC, and COSY spectra were obtained with 25% NUS of 1024 F1 points.

The 1D ^{1}H VT set of the solution of PROTAC 2dissolved in DMSO-d6 was acquired using a Bruker 5 mm cryoprobe at a magnetic field of 11.7 T corresponding to a ^{1}H frequency of 500 MHz. The temperature was controlled using a 400 lph VT flow. The time allowed for temperature equilibration between subsequent spectra was 10 minutes.

Table S3. Experimental details for the VT measurements of PROTAC 2 dissolved in DMSO-d_6.

Experiment	T_{set} (K)	^{1}H 90° RF amplitude (kHz)	recycle delay(s)	Number of FID points	SW (kHz)	Size of real spectrum	Number of scans
1D ^{1}H pulse-acquire	293	35	1	65536	10	65536	16
1D ^{1}H pulse-acquire	298	35	1	65536	10	65536	16
1D ^{1}H pulse-acquire	308	35	1	65536	10	65536	16
1D ^{1}H pulse-acquire	318	35	1	65536	10	65536	16

2. Additional NMR data

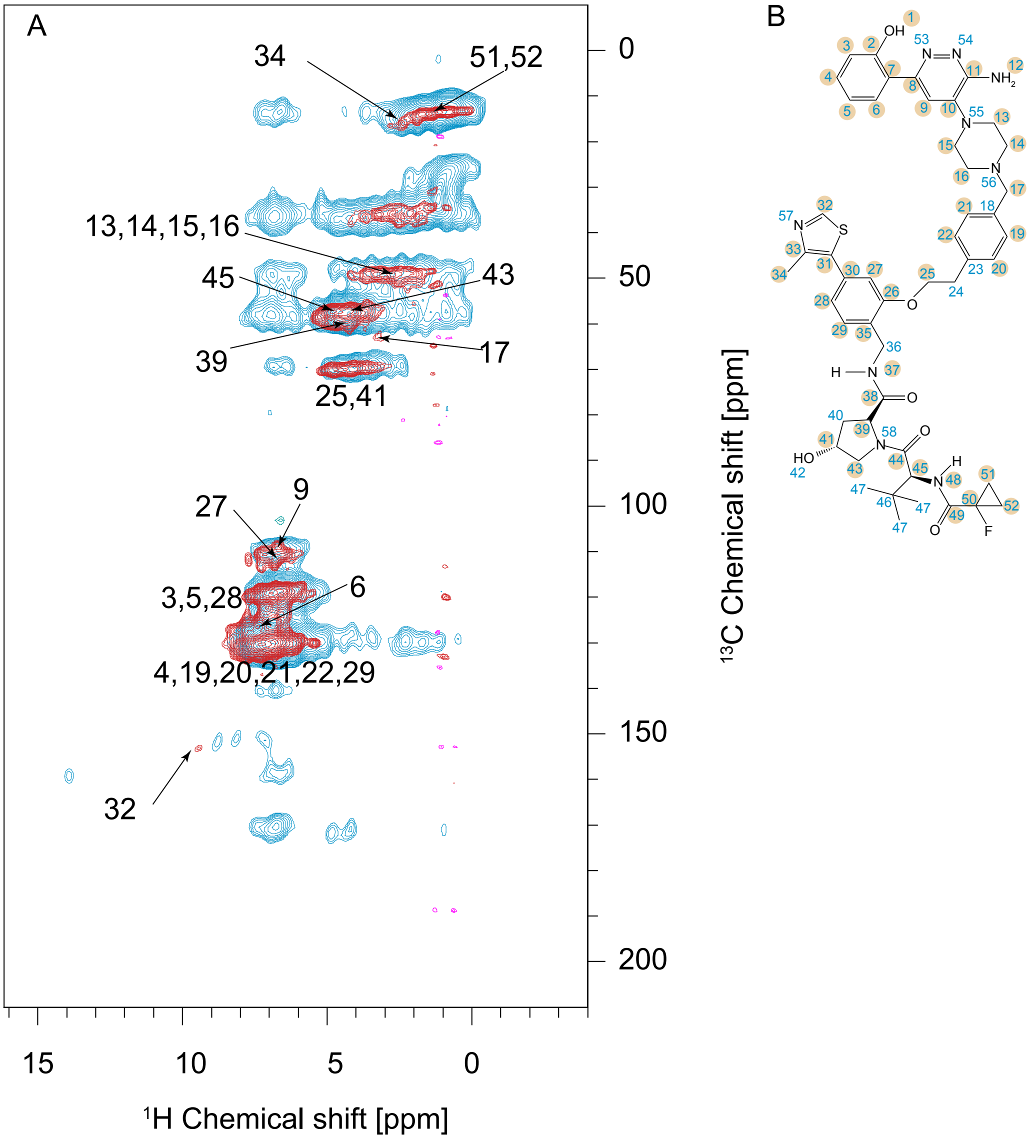

Figure S1. A) 900 MHz 1D ^{1}H spectrum of the as-received powder of PROTAC 2 acquired at 293 K with 100 kHz MAS enlarged for clarity in spectral assignment. **B)** Chemical structure of PROTAC 2 with the labelling scheme used here. The labels of atoms whose experimental chemical shift distributions are used for comparison with predicted shifts are highlighted by orange circles.

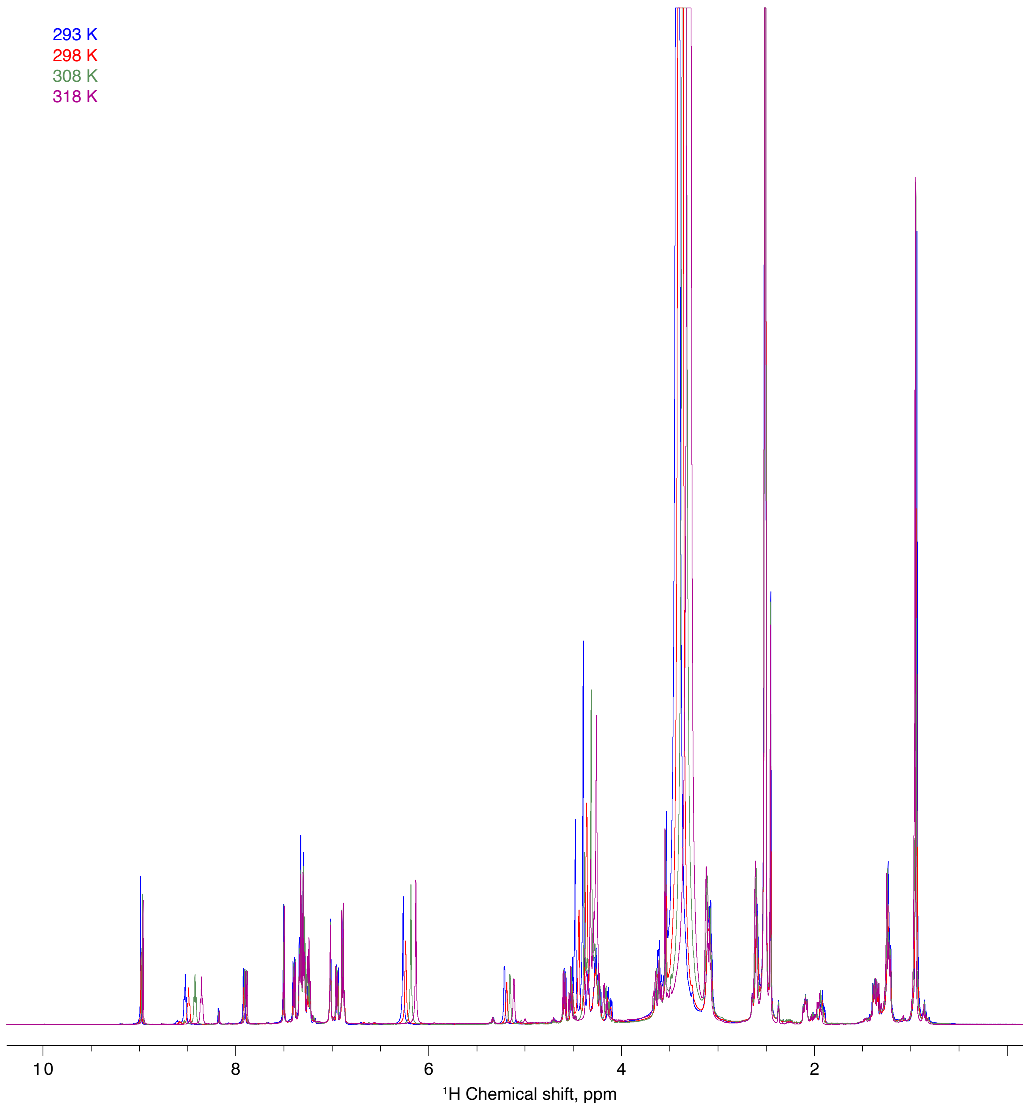

Figure S2. 1D ^{1}H spectra of a solution of PROTAC 2 dissolved in DMSO-d6 acquired at variable sample temperature (indicated next to the spectra)

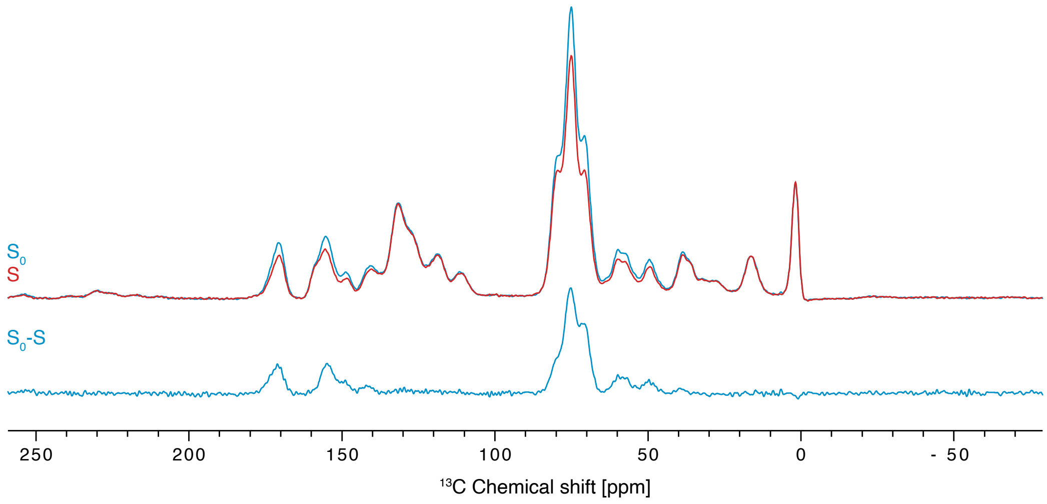

Figure S3. Attached ^{14}N test of the frozen solution of PROTAC 2 with 1.4 ms recoupling time: ^{13}C{^{14}N} RESPDOR spectrum without the ^{14}N pulse (S_0); ^{13}C{^{14}N} RESPDOR spectrum with the ^{14}N pulse (S) and the difference between the two (S_0-S).

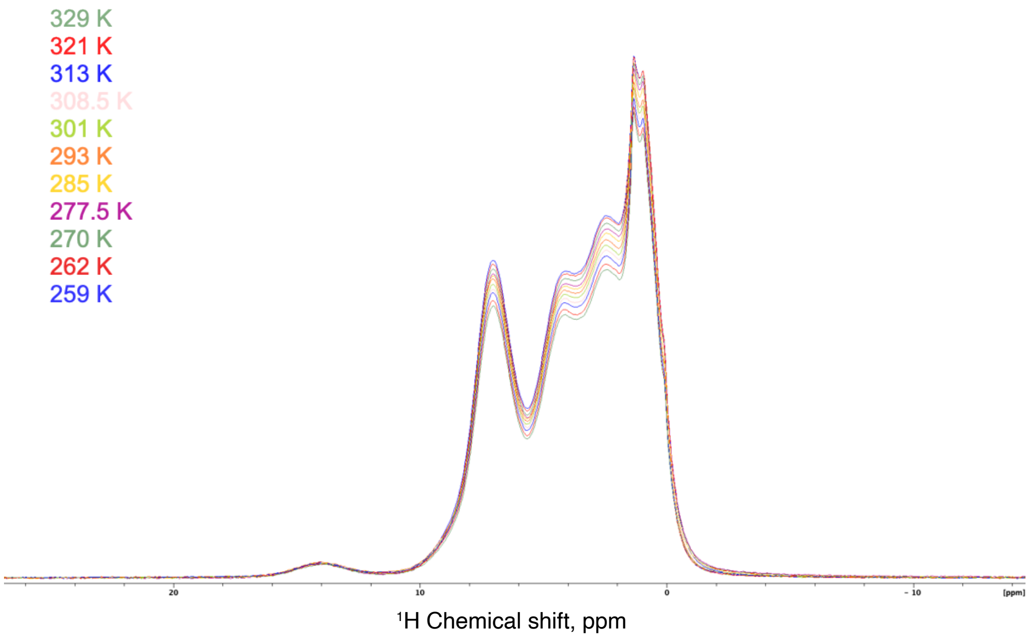

Figure S4. 1D ^{1}H spectra of the as-received powder of PROTAC 2 acquired at variable sample temperature (indicated next to the spectra)

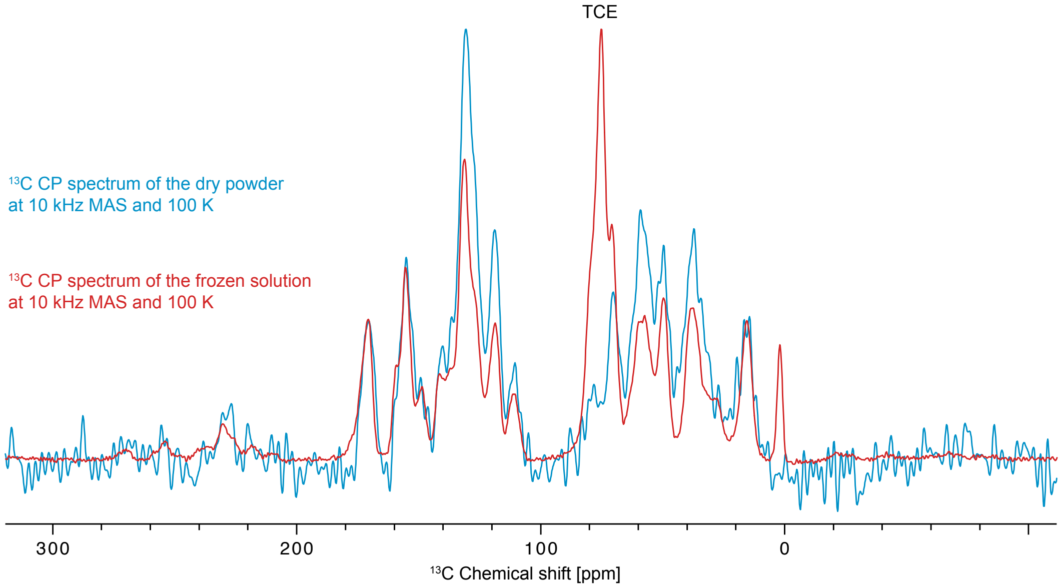

Figure S5. Comparison of ^{13}C spectra for the frozen-solution and the dry powder of the as-receive PROTAC 2.

3. Assignment and fitting details

Table S4. Solution-state NMR assignment of ^{1}H and ^{13}C chemical shifts of PROTAC 2 dissolved in DMSO-d6 at room temperature.

Atom label	^{13}C Chemical Shift, ppm	^{15}N Chemical Shift, ppm	^{1}H Chemical Shift, ppm
1			4.43
2	159.0		
3	117.8		6.88
4	130.6		7.24
5	118.9		6.88
6	126.6		7.91
7	118.2		
8	155.1		
9	110.8		7.5
10	153.6		
11	140.9		
13	49.0		3.10 / 3.10
12		69.1	6.23 / 6.23
14	52.4		2.59 / 2.59
15	49.0		3.10 / 3.10
16	52.4		2.59 / 2.59
17	62.2		3.53 / 3.53
18	136.4		
19	129.4		7.33
20	129.3		7.29
21	129.4		7.33
22	129.3		7.29
23	137.8		
24	35.2		3.07 / 3.07
25	69.0		4.27 / 4.27
26	156.2		
27	112.1		7.02
28	121.3		6.95
29	128.1		7.39
30	131.7		
31	131.3		
32	151.2		8.99
33	148.3		
34	16.4		2.45
35	127.3		
36	37.7		4.14 / 4.24
37		110.1	8.48
38	172.3		
39	59.2		4.51
40	38.3		2.09 / 1.91
41	69.3		4.34
42			5.18
43	57.1		3.61 / 3.62
44	169.4		
45	57.0		4.59
46	36.5		
47	26.6		0.94
48		112.0	7.29
49	168.6		
50	78.6		
51	13.4		1.21 / 1.37
52	13.1		1.21 / 1.35

Table S5. ^{13}C and ^{1}H mean chemical shifts (μ) and linewidths (σ) obtained by fitting solid-state NMR spectra of the as received powder of PROTAC 2.

Atom label	^{13}C μ, ppm	^{13}C σ, ppm	^{1}H μ, ppm	^{1}H σ, ppm
1			13.9	1.4
2	159.3	1.1		
3	119.2	1.5	6.9	0.7
4	130.6	1.8	7.1	0.7
5	119.2	1.5	6.9	0.7
6	125.4	1.7	7.3	0.7
7	117.7	1.7		
8	155.1	1.8		
9	108.4	1	6.8	0.7
10	155.1	1.8		
11	141.7	1.4		
13	49.4	1.9	2.8 / 2.8	1.1 / 1.1
12			5.2 / 6.9	0.3 / 0.8
14	49.4	1.9	2.8 / 2.8	1.1 / 1.1
15	49.4	1.9	2.8 / 2.8	1.1 / 1.1
16	49.4	1.9	2.8 / 2.8	1.1 / 1.1
17	63.1	1.7	3.2 / 3.2	0.8 / 0.8
18	137.0	3.0		
19	130.6	1.8	7.1	0.7
20	130.6	1.8	7.1	0.7
21	130.6	1.8	7.1	0.7
22	130.6	1.8	7.1	0.7
23				
24				
25	70	1.1	4.3 / 4.3	0.8 / 0.8
26	155.1	1.8		
27	111.6	1.9	6.8	0.7
28	119.2	1.5	6.8	0.6
29	130.6	1.8	7.1	0.7
30	131.9	1.5		
31	131.9	1.5		
32	152.7	1.6	9.1	1.0
33	148.4	1.7		
34	15.6	1.5	2.3	0.5
35	127.3	3.0		
36				
37			8.0	1.2
38	170.7	1.8		
39	60.0	1.3	4.6	0.8
40				
41	70.0	1.1	4.3	0.8
42				
43	57.2	1.2	4.5 / 4.5	0.8 / 0.8
44	170.7	2.0		
45	57.5	1.2	4.9	0.3
46				
47				
48			8.0	1.2
49	170.7	1.8		
50	80.6	1.2		
51	13.6	0.8	1.1 / 1.1	0.8 / 0.8
52	13.6	0.8	1.1 / 1.1	0.8 / 0.8

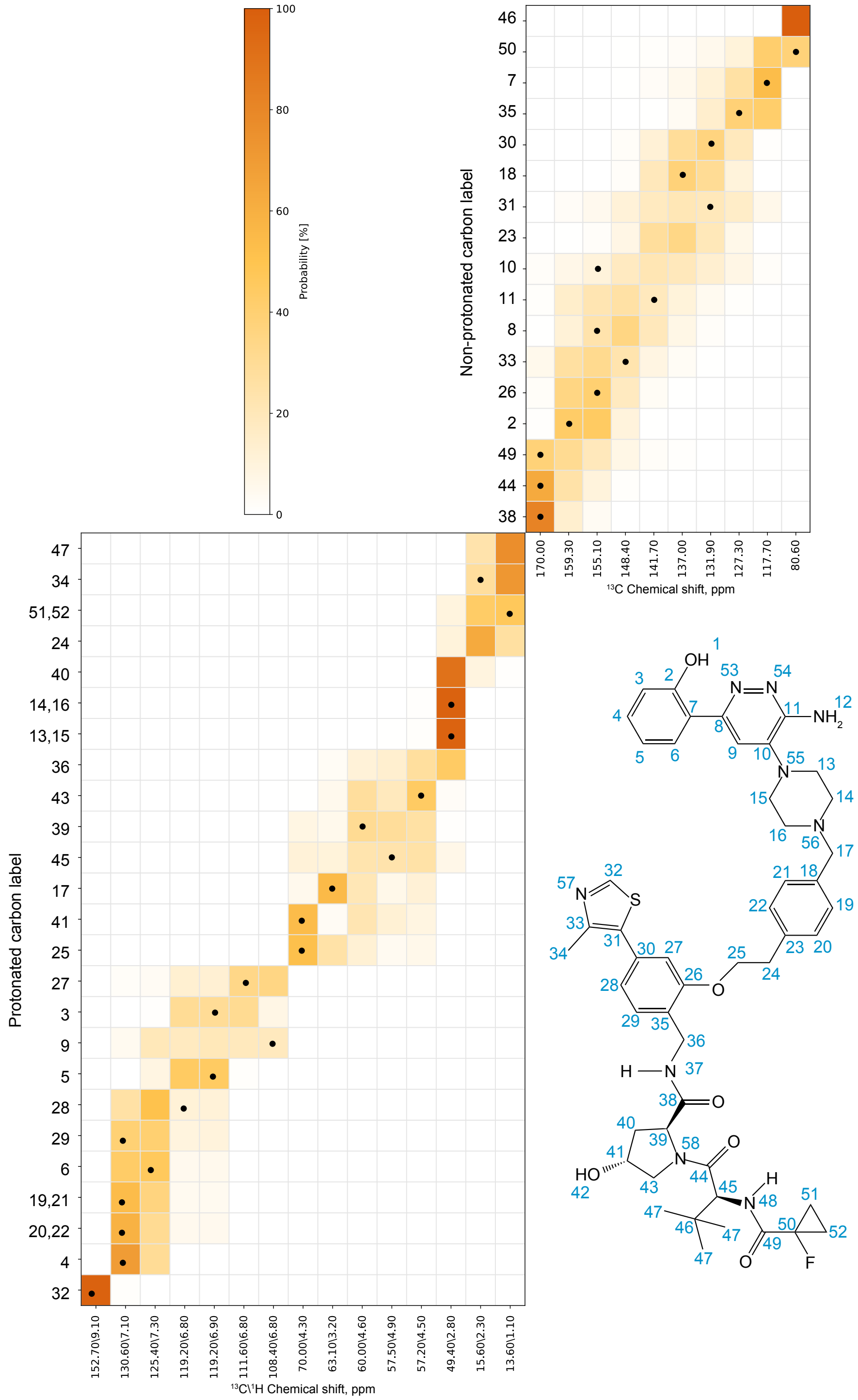

Figure S6. Prior individual assignment probabilities of the ^{13}C (and ^{1}H) chemical shifts after Bayesian inference of the global assignments of the protonated and quaternary carbons and the chemical structure of PROTAC 2 with the labelling scheme. The dots indicate the experimentally determined correct assignment.

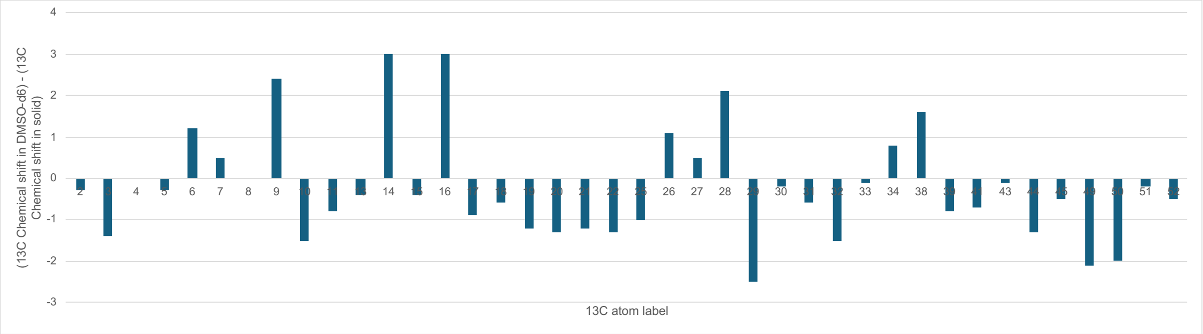

Figure S7. Variation of ^{13}C chemical shift between solution- and solid-state NMR per atom.

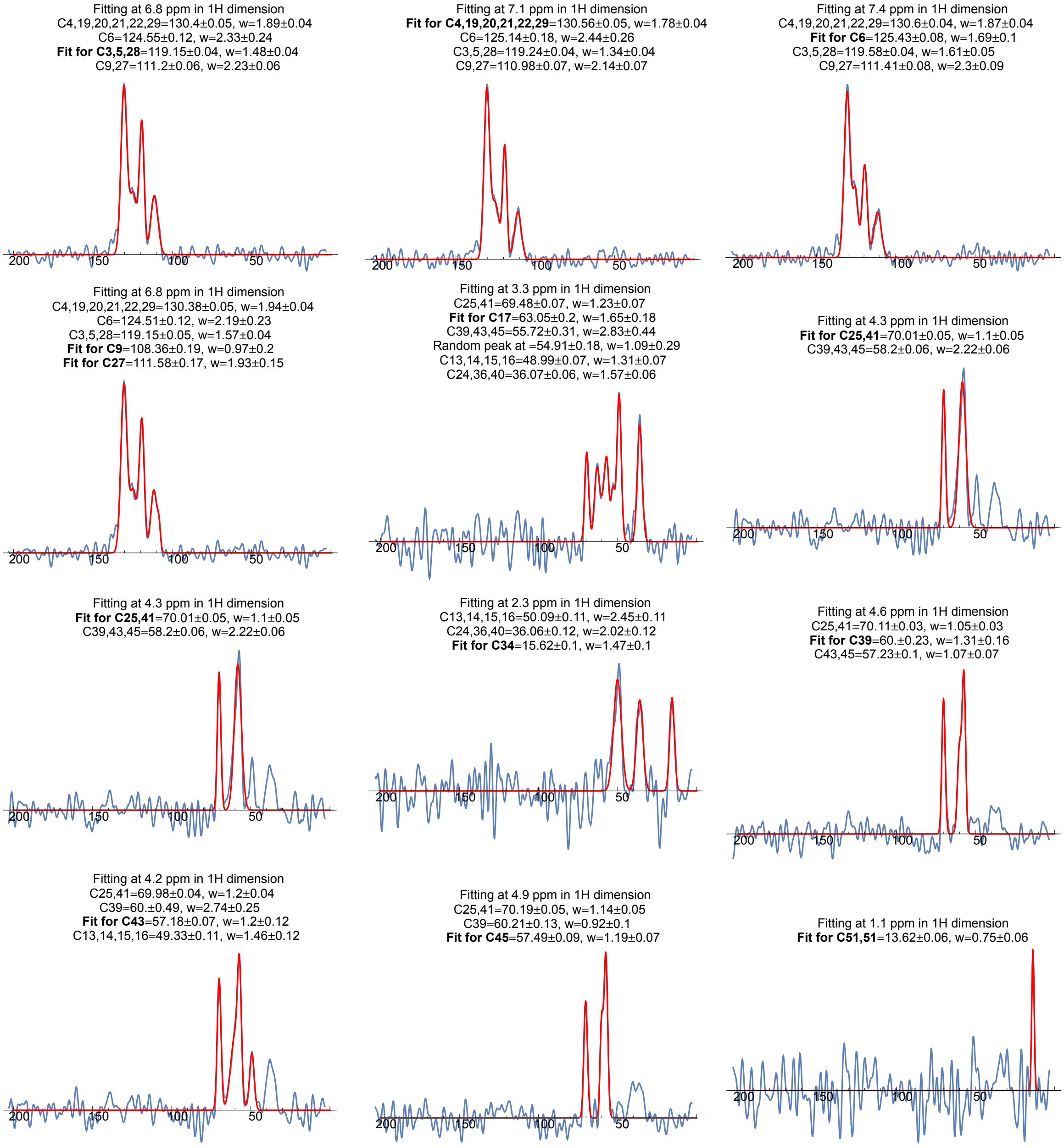

Figure S8. Fitting results of ^{13}C peaks extracted from hCH columns to Gaussian functions.

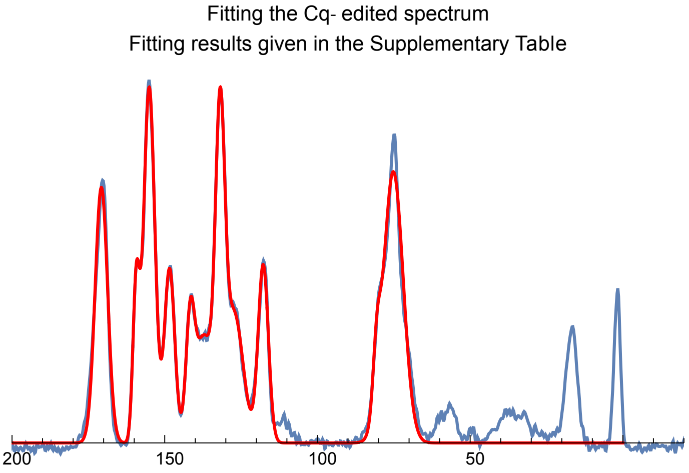

Figure S9. Fitting results of ^{13}C peaks from C_q-edited spectrum to Gaussian functions. Fitting results are given in Table S5.

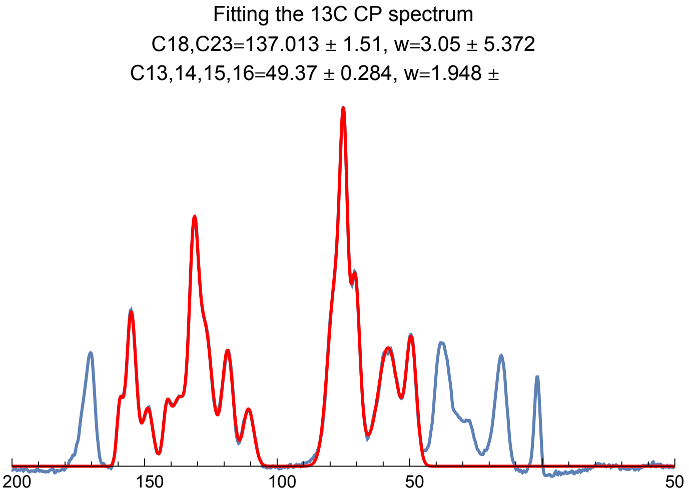

Figure S10. Fitting results of ^{13}C peaks from ^{13}C CP columns to Gaussian functions.

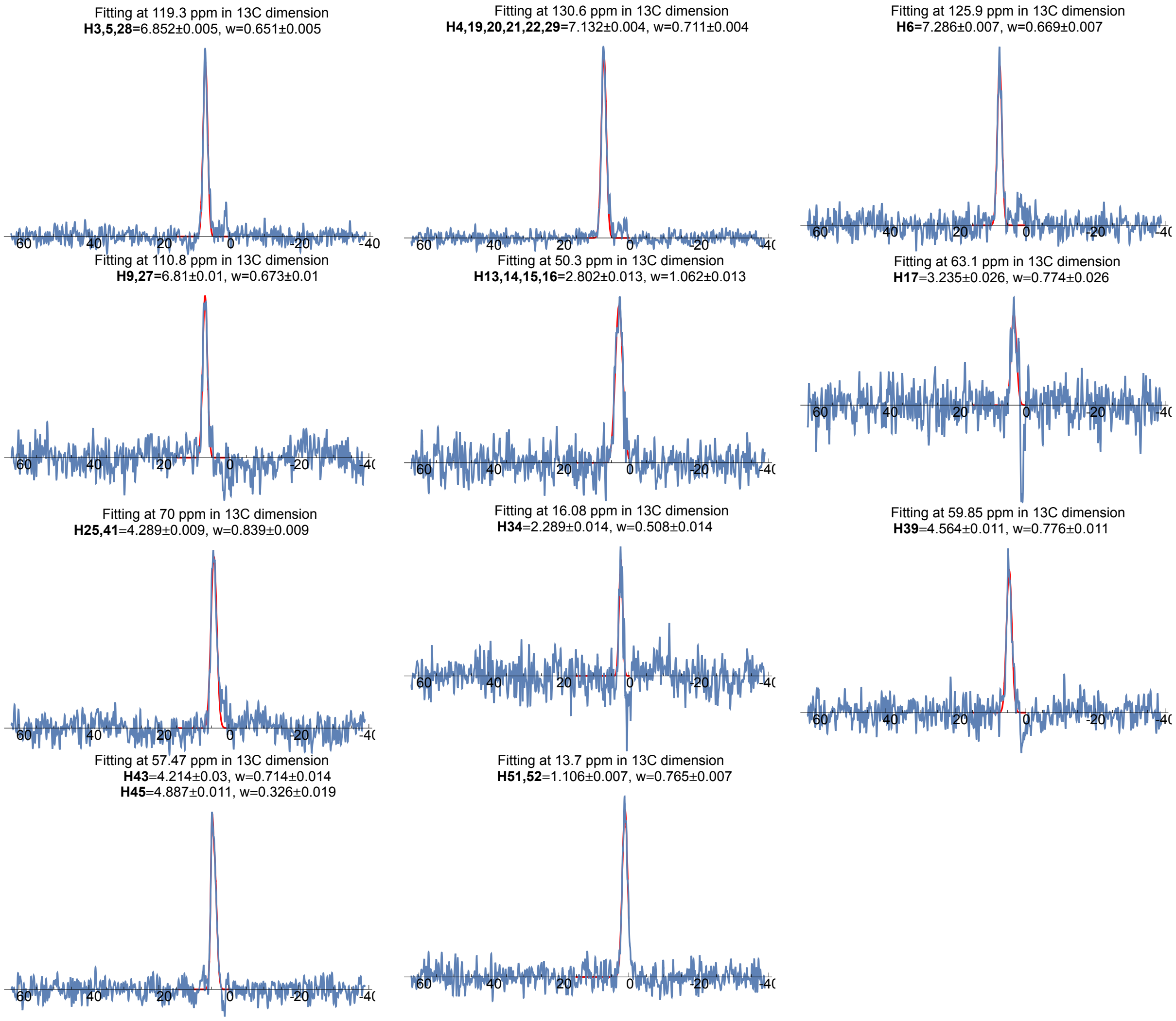

Figure S11. Fitting results of ^{1}H peaks extracted from hCH rows to Gaussian functions.

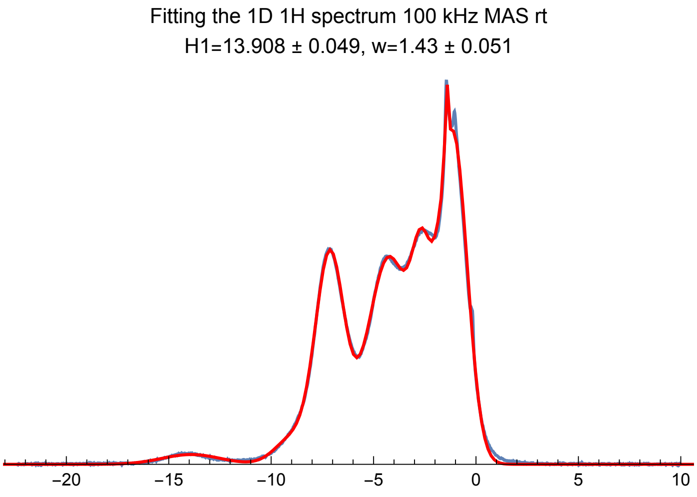

Figure S12. Fitting results of H1 peak extracted from the 1D ^{1}H spectrum at 100 kHz MAS rt.

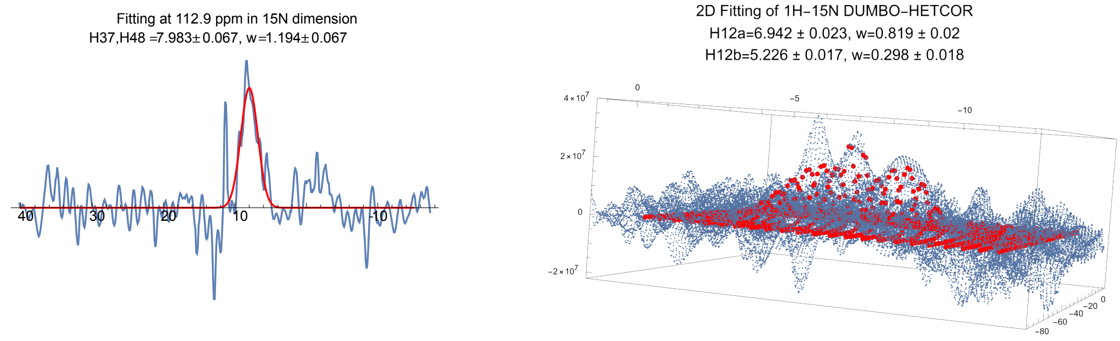

Figure S13. Fitting results of NH ^{1}H peaks from ^{1}H-^{15}N DUMBO-HETCOR columns to Gaussian functions.

4. Additional histograms of the dihedral angles and relative cluster formation energies

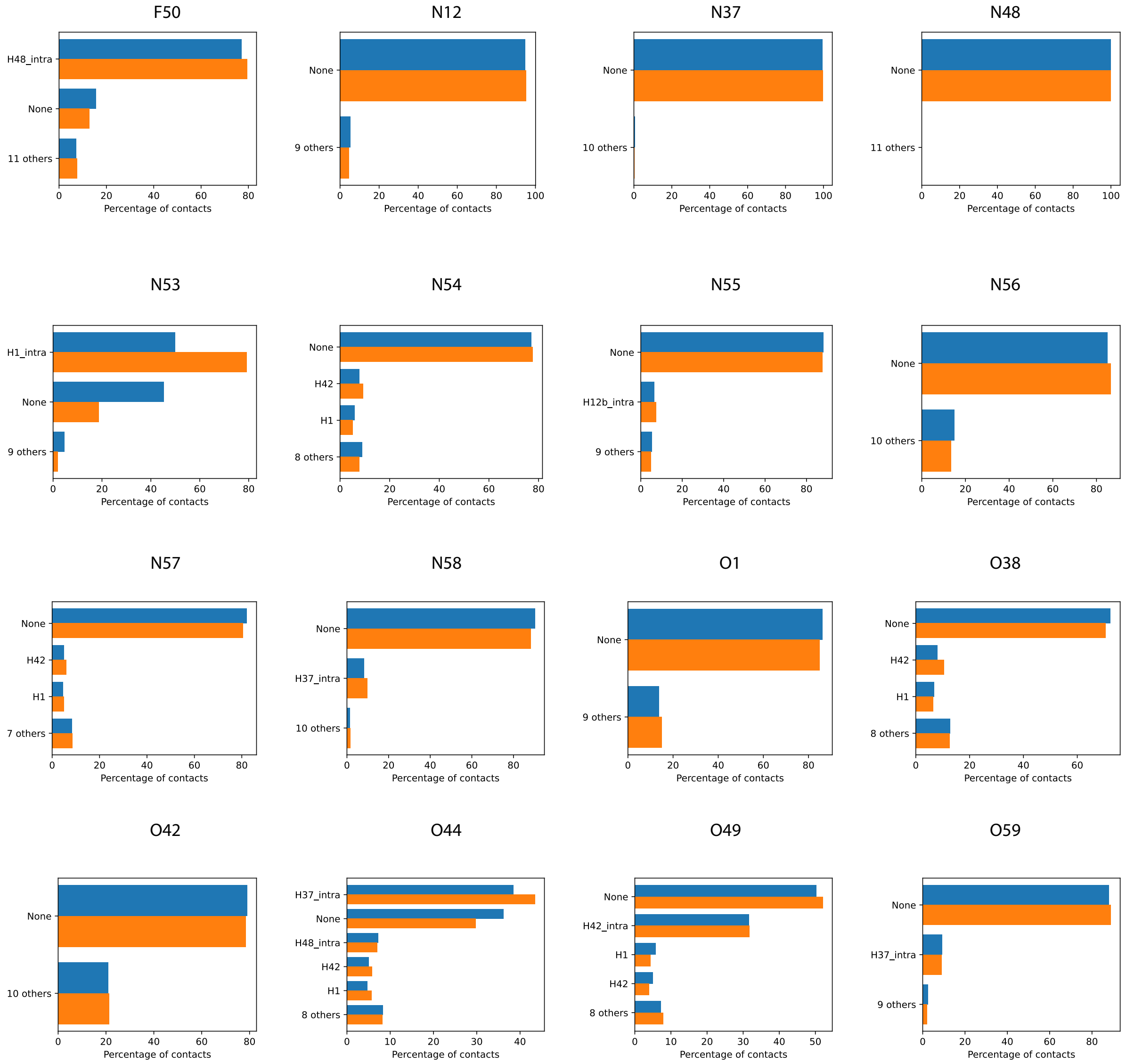

Figure S14. Occurrence of H-bonding for different H-bonding acceptors indicated above each panel in the MD set (blue) and NMR set (orange).

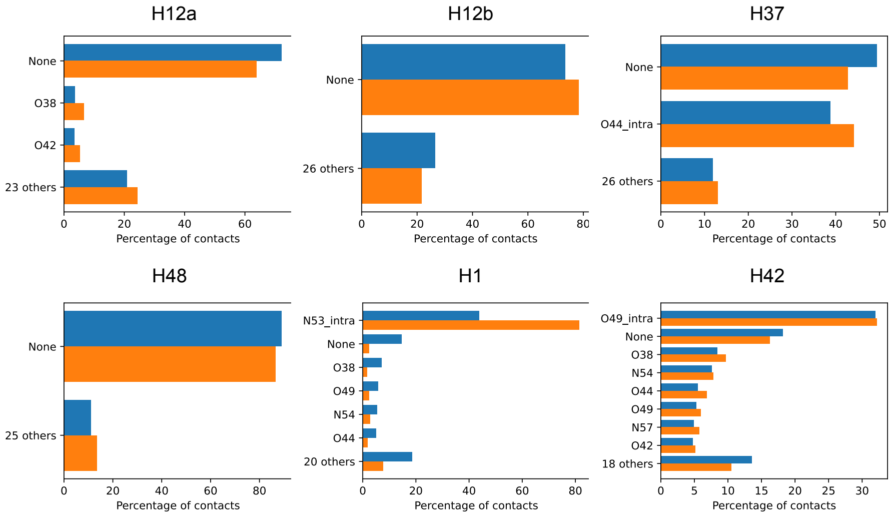

Figure S15. Occurrence of H-bonding for different H-bonding donors indicated above each panel in the MD set (blue) and NMR set (orange).

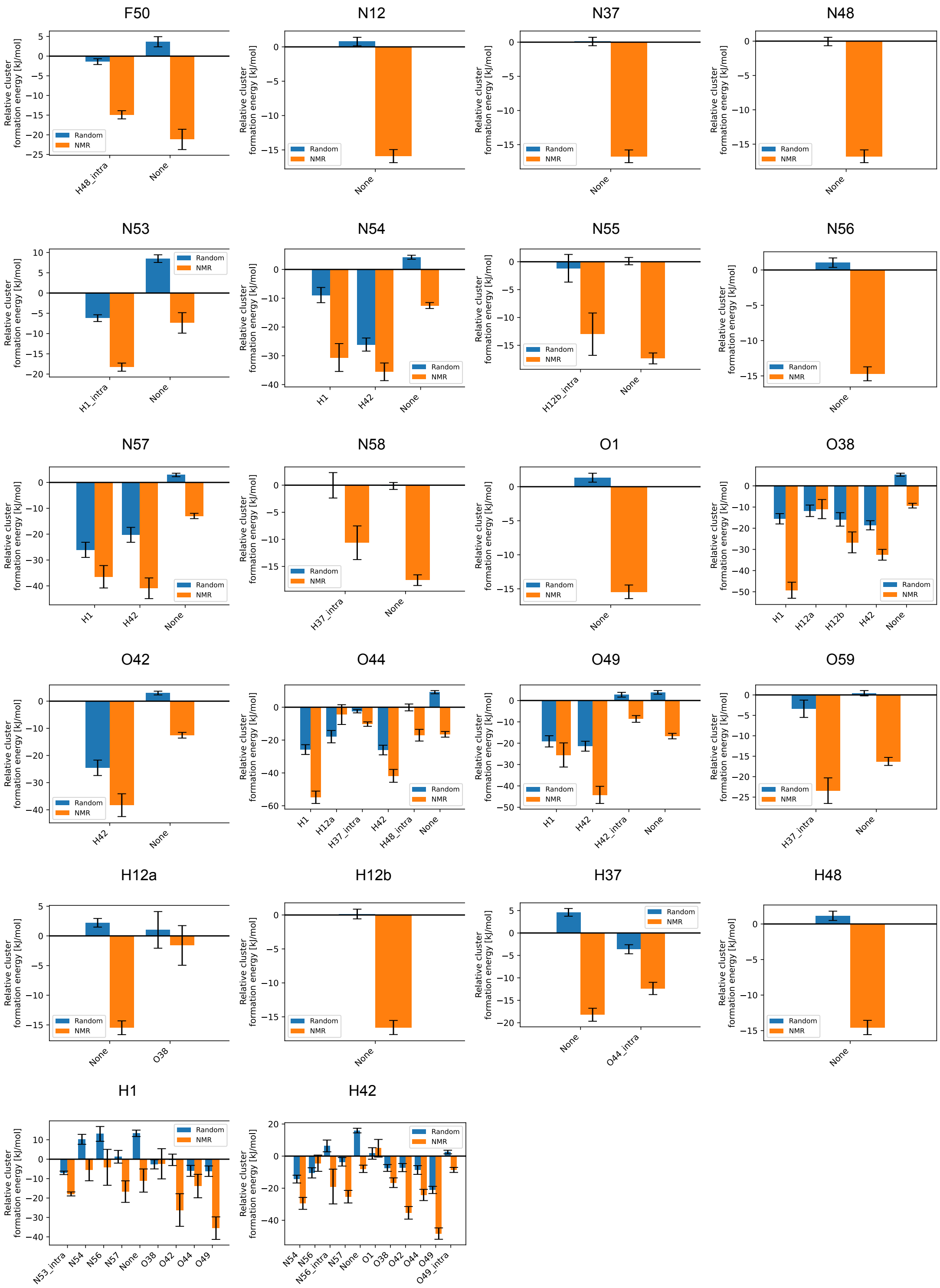

Figure S16. Relative formation energies of the local molecular environments in 12,000 structures randomly selected from the MD set (blue) and the 5,000 structures contained in the NMR set (orange) for different H-bond acceptors and donors indicated above each panel as a function of H-bonding partner. Error bars represent the standard error of the mean relative formation energy.

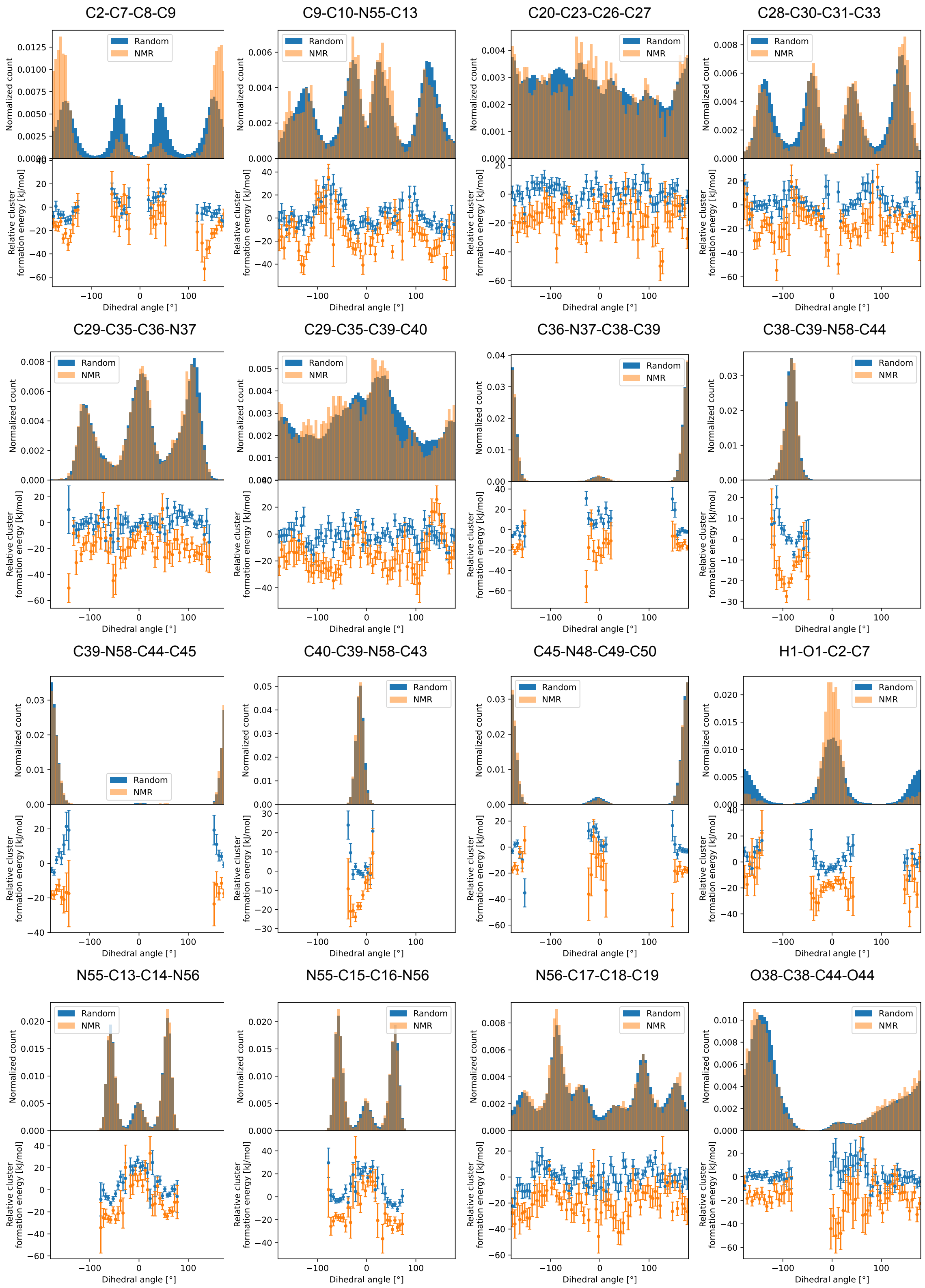

Figure S17. Histograms of the dihedral angles indicated above each panel in the MD set (blue) and the NMR set (orange) and the relative formation energies of the local molecular environments in 12,000 structures randomly selected from the MD set (blue) and the 5,000 structures contained in the NMR set (orange) as a function of the dihedral angle value.

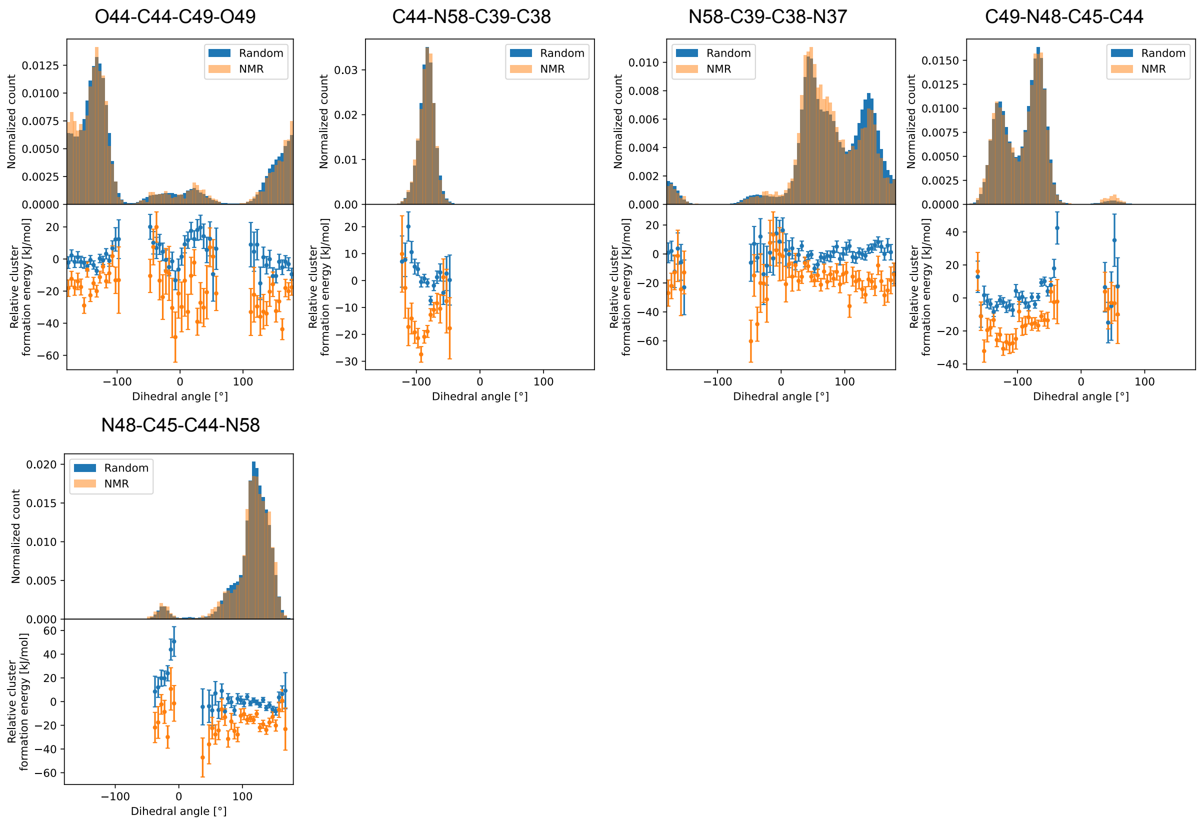

Figure S17 (continuation). Histograms of the dihedral angles indicated above each panel in the MD set (blue) and the NMR set (orange) and the relative formation energies of the local molecular environments in 12,000 structures randomly selected from the MD set (blue) and the 5,000 structures contained in the NMR set (orange) as a function of the dihedral angle value.

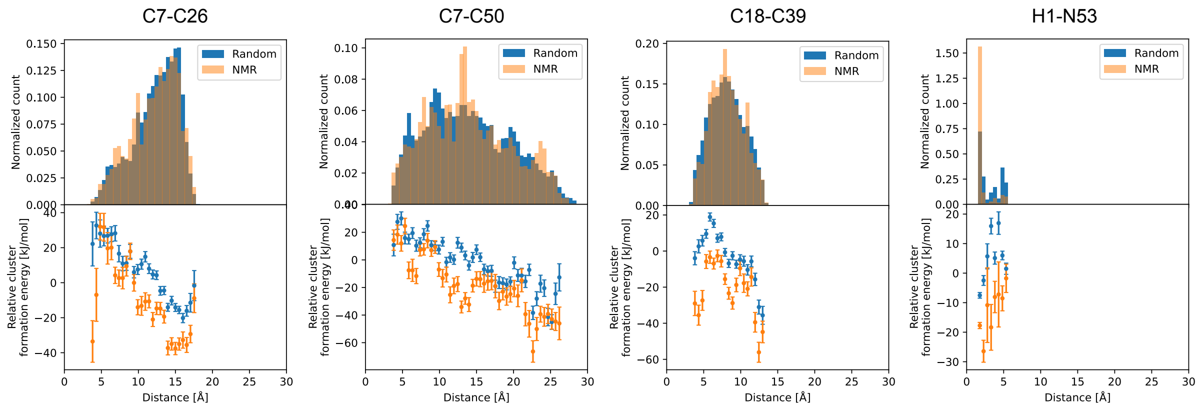

Figure S18. Histograms of the interatomic distances between pairs of atoms indicated above each panel in the MD set (blue) and the NMR set (orange) and the relative formation energies of the local molecular environments in 12,000 structures randomly selected from the MD set (blue) and the 5,000 structures contained in the NMR set (orange) as a function of the distance.

5. Computational details for the MD simulations

Computational details for the MD simulations: To model the amorphous structure of PROTAC 2, we carried out MD simulations on periodic amorphous cells. To the atomic positions of a single molecule extracted from PDBID 6HAX hydrogens were added with the Mercury software. This structure was then optimized at the B3LYP-D3/6-31G(d,p) level of theory in gas phase using the Gaussian 16 revision C.01 program. Coordinates and CHELPG charges were extracted and used as input to generate amorphous cells. Materials Studio 2020 employing the COMPASS-III force field was used to create cubic amorphous cells of 128 molecules placed randomly in five replicates. Three additional starting conformations were created by manually modifying the two trans amide bond conformations in the experimental structure into cis amide conformations, either as mono-modified or as doubly modified. These multiple replicas allow the generation of a diverse set of MD-structures. PDB files of the amorphous cells were saved as input for the MD-step. The Desmond program (Schrödinger 2021-4) was used for all MD simulations throughout the study employing the OPLS4 force field. The systems were initially equilibrated for 1 ns using the canonical (NVT) ensemble first at 100K and then at 298K. The temperature was held constant using a Nosé-Hoover chain thermostat with a relaxation time of 1.0 ps. A second equilibration was carried out for 32 ns using the isothermal-isobaric ensemble (NPT) at 298K and 1 bar where the temperature and pressure were held constant using the coupled Martyna-TobiasKlein method with a relaxation time of 1.0 ps. Production simulations were carried out for 750 ns using the NPT ensemble at 298K and 1 bar with the same settings as in the second equilibration. Electrostatic interactions were included with a 9 Å cutoff. Trajectories were collected every 100 ps. Models of the amorphous structure were obtained by extracting evenly spaced snapshots from the last 100 ns of each MD simulation. The raw MD data is uploaded to AstraZeneca.

6. Synthesis of PROTAC 2

Complete reaction scheme for the synthesis of PROTAC 2

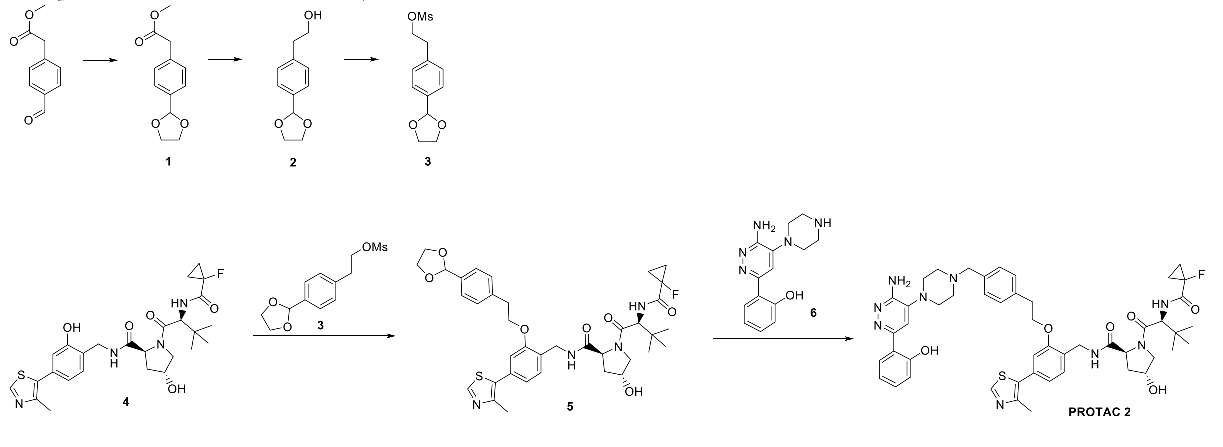

4-(1,3-dioxolan-2-yl)phenethyl methanesulfonate (**3**)

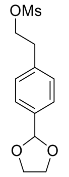

Tetra-n-butylammonium tribromide (0.812 g, 1.68 mmol) was added to methyl 2-(4-formylphenyl)acetate (15 g, 84.18 mmol) in ethylene glycol (25 mL) and triethyl orthoformate (20 mL). The resulting mixture was stirred at 25 °C for 18 hours in the dark. The reaction mixture was diluted with EtOAc (25 mL). The organic layers were combined and washed with water (2 x 20 mL) and brine (20 mL). The organic layer was dried over Na_2SO_4, filtered and evaporated to afford crude product. The crude product was purified by flash silica chromatography, elution gradient 0 to 100% EtOAc in petroleum ether. The pure fractions were evaporated to dryness to afford methyl 2-(4-(1,3-dioxolan-2-yl)phenyl)acetate (**1**) (12.00 g, 64.1 %) as a colourless oil.

2.5 M $LiAlH_4$ in THF (21.6 mL, 54.0 mmol) was added to methyl 2-(4-(1,3-dioxolan-2-yl)phenyl)acetate (**1**) (6 g, 27.00 mmol) in THF (60 mL) cooled to 0°C under nitrogen. The resulting mixture was stirred at 25 °C for 18 hours. The reaction mixture was sequentially treated with water (50 mL), 2M aqueous sodium hydoxide (2 x 50 mL) and water (2 x 50 mL). The mixture was diluted with EtOAc (100 mL), the organic layer was dried over Na_2SO_4, filtered and evaporated to afford 2-(4-(1,3-dioxolan-2-yl)phenyl)ethan-1-ol (**2**) (5.00 g, 95 %) as a colourless oil, which was used in the next step without any further purification.

Methanesulfonic anhydride (6.05 g, 34.75 mmol) was added to 2-(4-(1,3-dioxolan-2-yl)phenyl)ethan-1-ol (**2**) (4.50 g, 23.17 mmol) and triethylamine (16.15 mL, 115.8 mmol) in CH_2Cl_2 (50 mL) cooled to 0°C over a period of 1 minute under nitrogen. The resulting solution was stirred at 0 °C for 30 minutes. The solvent was removed under reduced pressure to afford crude product. The crude product was purified by flash silica chromatography, elution gradient 0 to 100% EtOAc in petroleum ether. Pure fractions were evaporated to dryness to afford 4-(1,3-dioxolan-2-yl)phenethyl methanesulfonate (**3**) (4.80 g, 76 %) as a colourless oil.

^{1}H NMR (400 MHz, $CDCl_3$) δ (ppm) 2.88 (s, 3H), 3.09 (t, J = 6 Hz, 2H), 4.00 – 4.21 (m, 4H), 4.43 (t, J = 8 Hz, 2H), 5.81 (s, 1H), 7.28 (d, J = 8 Hz, 2H), 7.46 (d, J = 8 Hz, 2H).

MS: $C_{12}H_{16}O_5S$; calcd. for $(M+H^+)$: 273.1, found: 273.2

(2S,4R)-N-(2-(4-(1,3-dioxolan-2-yl)phenethoxy)-4-(4-methylthiazol-5-yl)benzyl)-1-((S)-2-(1-fluorocyclopropane-1-carboxamido)-3,3-dimethylbutanoyl)-4-hydroxypyrrolidine-2-carboxamide (**5**)

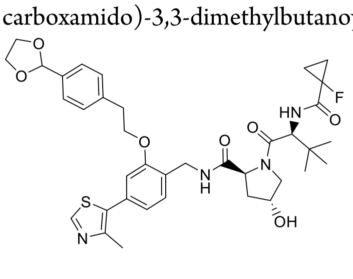

4-(1,3-dioxolan-2-yl)phenethyl methanesulfonate **(3)** (767 mg, 2.82 mmol) was added to (2S,4R)-1-((S)-2-(1-fluorocyclopropane-1-carboxamido)-3,3-dimethylbutanoyl)-4-hydroxy-N-(2-hydroxy-4-(4-methylthiazol-5-yl)benzyl)pyrrolidine-2-carboxamide **(4)** (500 mg, 0.94 mmol) and K_2CO_3 (389 mg, 2.82 mmol) in DMF (8 mL). The resulting mixture was stirred at 70 °C for 14 hours. The resulting reaction mixture was diluted with water (20 mL) and CH_2Cl_2 (40 mL), and subsequently extracted with CH_2Cl_2 (3 x 25 mL). The organic layer was dried over Na_2SO_4, filtered and evaporated to afford crude product. The crude product was purified by flash silica chromatography, elution gradient 0 to 10% MeOH in CH_2Cl_2. The pure fractions were evaporated to dryness to afford (2S,4R)-N-(2-(4-(1,3-dioxolan-2-yl)phenethoxy)-4-(4-methylthiazol-5-yl)benzyl)-1-((S)-2-(1-fluorocyclopropane-1-carboxamido)-3,3-dimethylbutanoyl)-4-hydroxypyrrolidine-2-carboxamide **(5)** (480 mg, 72.1 %) as a colourless oil.
^{1}H NMR (400 MHz, DMSO-d_6) δ (ppm) 0.97 (s, 9H), 1.19 – 1.26 (m, 2H), 1.34 – 1.41 (m, 2H), 1.88 – 2.03 (m, 1H), 2.05 – 2.15 (m, 1H), 2.46 (s, 3H), 3.10 (t, *J* = 6 Hz, 2H), 3.57 – 3.70 (m, 2H), 3.87 – 3.96 (m, 2H), 3.96 – 4.08 (m, 2H), 4.19 – 4.30 (m, 4H), 4.36 (s, 1H), 4.51 (t, *J* = 8 Hz, 1H), 4.60 (d, *J* = 6 Hz, 1H), 5.19 (s, 1H), 5.69 (s, 1H), 6.94 (d, *J* = 4 Hz, 1H), 6.99 – 7.04 (m, 1H), 7.28 – 7.34 (m, 1H), 7.35 – 7.40 (m, 4H), 7.64 – 7.76 (m, 1H), 8.46 – 8.54 (m, 1H), 8.99 (s, 1H).
MS: $C_{37}H_{45}FN_4O_7S$; calcd. for $(M+H^+)$: 709.3, found: 709.1

(2S,4R)-N-(2-(4-((4-(3-amino-6-(2-hydroxyphenyl)pyridazin-4-yl)piperazin-1-yl)methyl)phenethoxy)-4-(4-methylthiazol-5-yl)benzyl)-1-((S)-2-(1-fluorocyclopropane-1-carboxamido)-3,3-dimethylbutanoyl)-4-hydroxypyrrolidine-2-carboxamide **(PROTAC2)**

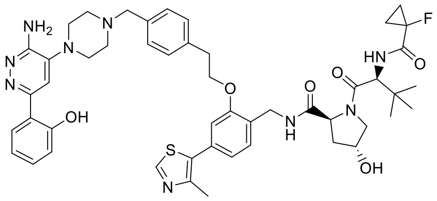

(2S,4R)-N-(2-(4-(1,3-dioxolan-2-yl)phenethoxy)-4-(4-methylthiazol-5-yl)benzyl)-1-((S)-2-(1-fluorocyclopropane-1-carboxamido)-3,3-dimethylbutanoyl)-4-hydroxypyrrolidine-2-carboxamide **(5)** (280 mg, 0.40 mmol) was dissolved in THF (5 mL) and 0.5 M hydrochloric acid aq (5 mL). The resulting solution was stirred at 50 °C for 1 hours. The solvent was removed under reduced pressure. The resulting mixture was taken up in 1,2-dichloroethane (15 mL) and DMSO (3 mL) before 2-(6-amino-5-(piperazin-1-yl)pyridazin-3-yl)phenol **(6)** (139 mg, 0.51 mmol), triethylamine (2.5 mL), $MgSO_4$ and Sodium triacetoxyborohydride (419 mg, 1.98 mmol) were added. The resulting mixture was stirred at 25 °C for 18 hours. The resulting reaction mixture was diluted with CH_2Cl_2 (15 mL) and MeOH (15 mL), filtered and concentrated under reduced pressure. The crude product was purified by C18-flash chromatography, elution gradient 0 to 100% MeCN in water. The pure fractions were evaporated to dryness to afford (2S,4R)-N-(2-(4-((4-(3-amino-6-(2-hydroxyphenyl)pyridazin-4-yl)piperazin-1-yl)methyl)phenethoxy)-4-(4-methylthiazol-5-yl)benzyl)-1-((S)-2-(1-fluorocyclopropane-1-carboxamido)-3,3-dimethylbutanoyl)-4-hydroxypyrrolidine-2-carboxamide (206 mg, 56 %) as a pink solid.

7. Solution ^{1}H-, ^{13}C- and ^{19}F-NMR, HRMS and X-ray diffraction data for PROTAC 2

(2S,4R)-N-(2-(4-((4-(3-amino-6-(2-hydroxyphenyl)24yridazine-4-yl)piperazin-1-yl)methyl)phenethoxy)-4-(4-methylthiazol-5-yl)benzyl)-1-((S)-2-(1-fluorocyclopropane-1-carboxamido)-3,3-dimethylbutanoyl)-4-hydroxypyrrolidine-2-carboxamide (**PROTAC 2**)

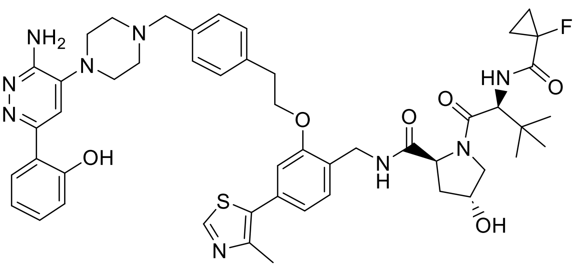

^{1}H NMR (500 MHz, DMSO-d_6) δ (ppm) 8.98 (s, 1H), 8.49 (t, *J* = 6.0 Hz, 1H), 7.93 – 7.88 (m, 1H), 7.50 (s, 1H), 7.39 (d, *J* = 7.8 Hz, 1H), 7.33 (d, *J* = 7.8 Hz, 2H), 7.28 (d, *J* = 8.0 Hz, 3H), 7.23 (td, *J* = 7.7, 1.6 Hz, 2H), 7.01 (d, *J* = 1.7 Hz, 1H), 6.94 (dd, *J* = 7.8, 1.6 Hz, 1H), 6.91 – 6.84 (m, 2H), 6.25 (s, 2H), 5.17 (d, *J* = 3.6 Hz, 1H), 4.58 (d, *J* = 9.2 Hz, 1H), 4.51 (t, *J* = 8.2 Hz, 1H), 4.34 (s, 1H), 4.30 – 4.20 (m, 3H), 4.12 (dd, *J* = 16.6, 5.6 Hz, 1H), 3.66 – 3.56 (m, 2H), 3.53 (s, 2H), 3.08 (dd, *J* = 13.4, 7.0 Hz, 4H), 2.59 (s, 3H), 2.45 (s, 3H), 2.09 (dd, *J* = 12.8, 7.8 Hz, 1H), 1.99 (dt, *J* = 15.8, 6.9 Hz, 1H), 1.91 (ddd, *J* = 13.1, 9.9, 4.5 Hz, 1H), 1.40 – 1.32 (m, 2H), 1.22 (dq, *J* = 11.3, 4.0 Hz, 4H), 0.94 (s, 9H).
^{13}C NMR (126 MHz, DMSO-d_6) δ (ppm) 172.3, 169.4, 168.5 (d, *J* = 20.4 Hz), 159.0, 156.1, 155.1, 153.6, 151.9, 148.3, 140.9, 137.8, 136.4, 131.7, 131.3, 130.6, 129.4 (2C), 129.3 (2C), 128.1, 127.4, 126.6, 121.3, 118.9, 118.2, 117.8, 112.1, 110.8, 79.5, 77.7, 69.4, 69.0, 62.2, 59.3, 57.2, 57.0, 52.4, 49.0, 38.4, 37.7, 36.5, 35.2, 29.6-29.0 (m) 26.6 (3C), 16.5, 13.5 (d, *J* = 10.1 Hz), 13.2 (d, *J* = 10.5 Hz).
^{19}F NMR (471 MHz, DMSO-d_6) δ (ppm)169.2 (s).
HRMS $C_{49}H_{58}FN_9O_6S$; calcd. For (M+H$^+$): 920.4288, found: 920.4293.

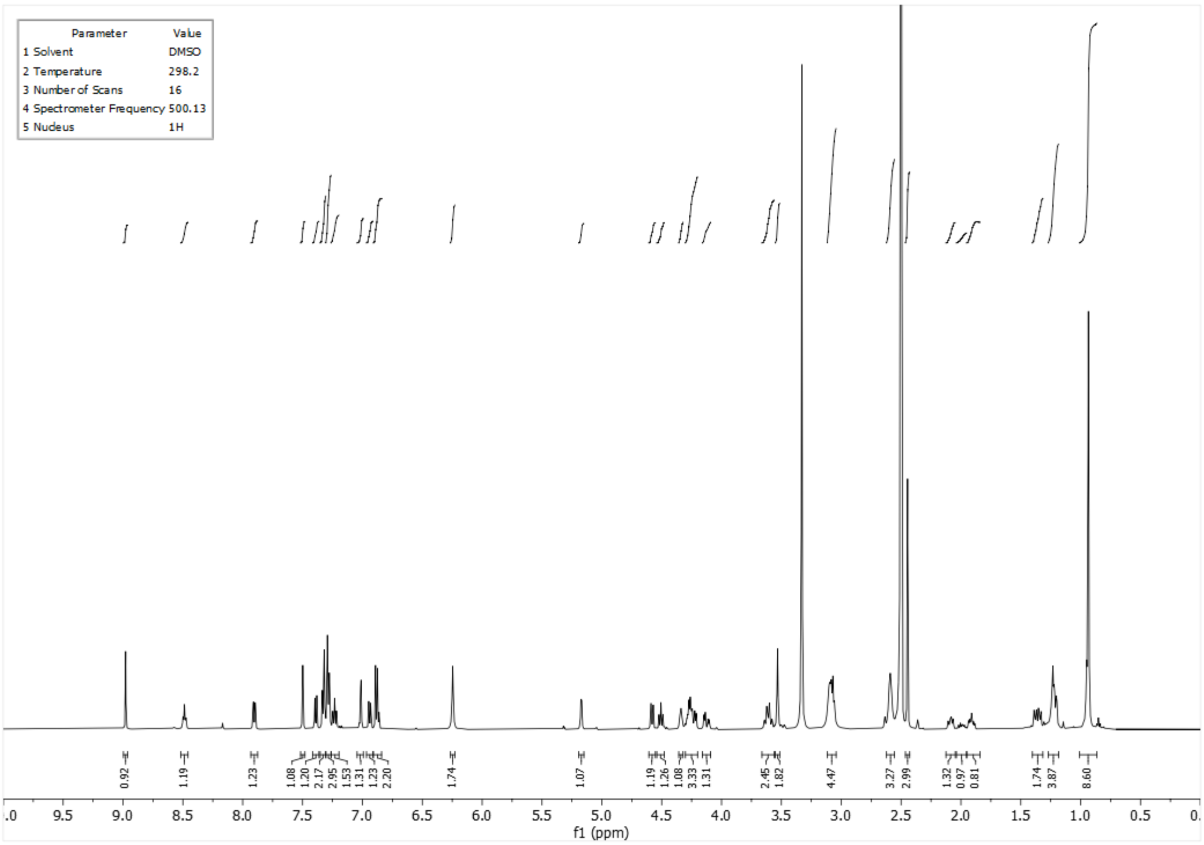

Figure S19. ^{1}H NMR spectrum

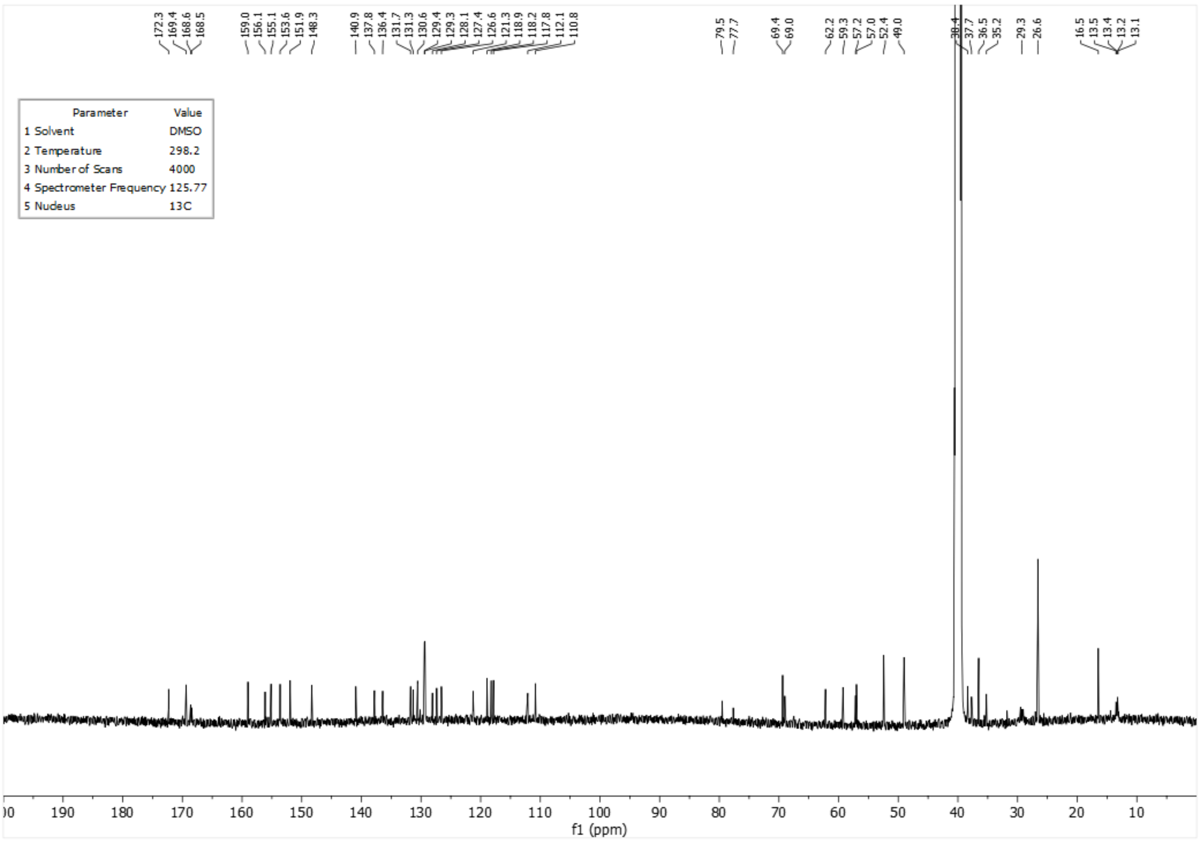

Figure S20. ^{13}C NMR spectrum

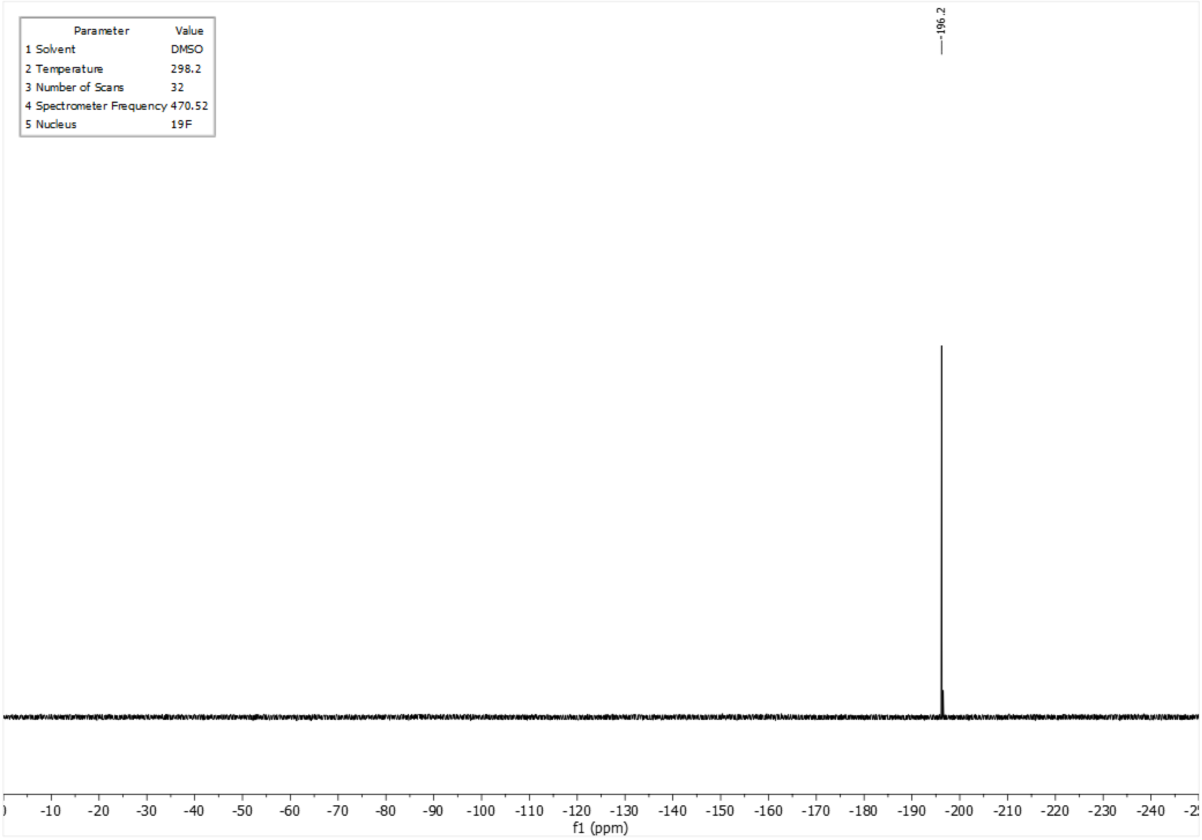

Figure S21. ^{19}F NMR spectrum

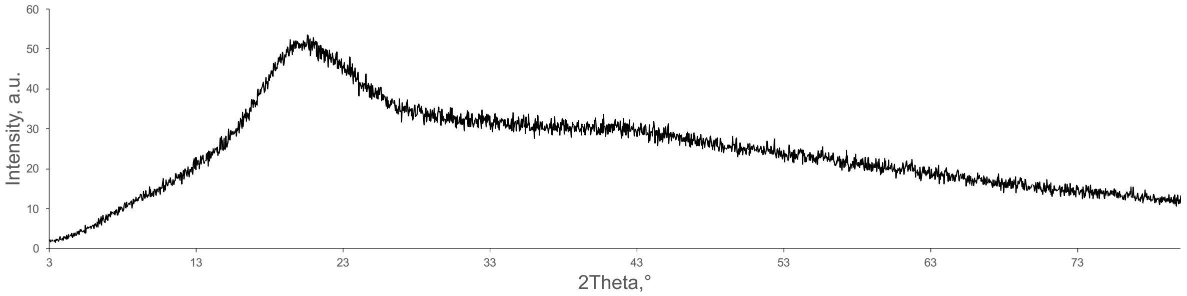

Figure S22. X-ray diffraction powder diffraction pattern of amorphous PROTAC 2
